# The impact of digital economy on the supply chain resilience of cross-border healthcare e-commerce

**DOI:** 10.3389/fpubh.2025.1570338

**Published:** 2025-05-23

**Authors:** Jianyu Liu, Siyi Mao, Lin Lu, Yuan Jing, Xiangjun Yang, Hejiang Xu, Yuelin Ren

**Affiliations:** ^1^School of Economics and Management, Guangxi Normal University, Guilin, China; ^2^Jinan Liangong Testing Technology Co., Ltd, Jinan, China; ^3^Key Laboratory of Digital Empowerment Economic Development, Guangxi Normal University, Guilin, China

**Keywords:** digital economy, healthcare industry, cross-border e-commerce, supply chain resilience, impact mechanisms

## Abstract

The healthcare industry plays a crucial role in global economic development and public health, but the healthcare cross-border e-commerce supply chain often faces numerous risks due to various disruptive events. We employ panel data from 2013 to 2022 for provincial-level regions in China to investigate the impact mechanisms of the digital economy on supply chain resilience in cross-border healthcare e-commerce. The results indicate that the digital economy significantly enhance supply chain resilience, with reduced reliance on foreign trade, increased export technology complexity, and decreased export concentration being key pathways for this improvement. The impact of the digital economy is stronger in the western regions than in central and eastern areas, and the establishment of comprehensive cross-border e-commerce pilot zones can further empower the supply chain. These findings offer valuable insights for the sustainable development of healthcare cross-border e-commerce supply chain resilience and the digital economy.

## 1 Introduction

The advent of digital technologies has fundamentally reconfigured conventional production, manufacturing, and operational paradigms, engendering the evolution of emergent industrial ecosystems and innovative development frameworks. The digital economy relies on the integration of data resources and digital technologies to promote economic development and enhance construction efficiency. The cross-border e-commerce supply chain in healthcare is an important application of the digital economy in the medical field, playing a vital role in global health initiatives. As digital technology and e-commerce continue to develop, the digital economy and healthcare cross-border e-commerce supply chain will further deepen and expand, injecting new vitality and momentum into global health efforts.

However, the instability and uncertainty of the VUCA environment pose challenges to the management of cross-border e-commerce supply chains. For example, the COVID-19 pandemic has revealed the vulnerability of supply chains due to shortages of medical supplies, highlighting the urgent need for improved management to achieve sustainable development. Although cross-border e-commerce supply chains are similar to traditional supply chains, they display diverse and decentralized characteristics influenced by the unique aspects of international trade.

As attention to supply chain resilience and risk management increases, researchers and practitioners are gradually focusing more on healthcare supply chains. Studies have explored resilience mechanisms from various perspectives, including the digital transformation of healthcare enterprises (1, 2) and lean management (3). However, there is still a lack of specific applications and theoretical research concerning the healthcare cross-border e-commerce supply chain. The complexity of the healthcare cross-border e-commerce supply chain and its significance in public health crises emphasize the necessity for further exploration and practical application.

This study centers on the core proposition of cross-border e-commerce development in the healthcare industry during the digital economy era, systematically addressing the critical scientific question of “how to develop adaptive mechanisms driven by digital technologies to enhance the resilience of cross-border medical supply chains.” Grounded in industrial and regional economics perspectives, the research deconstructs the multidimensional impact mechanisms of the digital economy on cross-border healthcare supply chains—encompassing three structural dimensions: foreign trade dependence, export technological sophistication, and export trade concentration—to establish an integrated analytical framework of “digital technologies–economic structures–supply chain synergies.” The potential contributions and innovations of this study are as follows: First, existing research primarily focuses on the impact of digital economy development on the socio-economic landscape, with an emphasis on its effects on domestic economic circulation. However, we focus on the resilience of healthcare cross-border e-commerce supply chains and explore the enabling mechanisms of the digital economy on external economic circulation. This approach will help to provide a more comprehensive assessment of the multidimensional impact of the digital economy on economic development. Second, this research is the first to establish an evaluation system for the resilience of healthcare cross-border e-commerce supply chains, contributing to the comprehensive theoretical framework related to supply chain resilience in the healthcare and cross-border e-commerce industries. Third, by analyzing the impact mechanisms of digital economy development on the resilience of healthcare cross-border e-commerce supply chains at the provincial level, this study can uncover the relational mechanisms between the digital economy and supply chain management. This enriches the theoretical research in both the digital economy and supply chain management fields and has significant practical and economic implications for promoting the development of the digital economy and the healthcare industry across different regions.

## 2 Literature review

### 2.1 Research of digital economy

#### 2.1.1 Definition of digital economy

The digital economy emerged as a new engine for global economic development in the 1990s. During this period, the rise of internet technology gave birth to e-commerce, with the emergence of industry giants like Amazon and eBay marking the embryonic form of the digital economy. Initially, the definition of the digital economy was confined to output related to the Information and Communication Technology (ICT) industry, representing the earliest narrow conception of the digital economy. As internet technologies became widely adopted across economic and social spheres, the U.S. Department of Commerce and OECD defined the digital economy as an integrated system encompassing IT production, application industries, and e-commerce. However, with the continuous advancement of new-generation information technologies, the essence and scope of the digital economy have been progressively expanding. Kling and Lamb (4) defined digital economic activities from the perspective of digital technology applications in the production, distribution, and exchange of products or services. Knickrehm and Daugherty (5) suggested that the portion of new additional output resulting from investments in information technology is termed the digital economy.

#### 2.1.2 Integration of digital economy and cross-border e-commerce

The digital economy has transcended traditional business models, inevitably impacting economic and social structures as well as enterprise operational paradigms (6, 7). Cross-border e-commerce, a novel application of international trade integrating information and communication technologies with internet technologies, has garnered significant attention globally. From the perspective of cross-border e-commerce supply chains, the digital economy also drives digitalization in supply chain management. Nunez-Merino et al. (8) emphasized the importance of enterprises leveraging digital technologies for continuous optimization and innovation in supply chain management within the context of the digital economy. Modern digital technologies enhance the capabilities for information sharing and collaboration in supply chains (9, 10), increase supply chain integration (11), and reduce negative value processes in supply chain economic activities (12). Numerous scholars have studied the role of digital technologies in empowering supply chains (13):suggested that the adoption of digital tools such as big data analytics can enhance the flexibility of product flows, thereby improving supply chain performance and resilience; Hazen et al. (14) and argued that big data analytics can enhance supply chain performance and drive innovation; Matthias et al. (15) also suggested that big data analytics can lower overall supply chain costs, enabling better decision-making and product and service offerings.

### 2.2 Research of supply chain resilience

#### 2.2.1 Definition of supply chain resilience

Rice and Caniato were among the first to propose the concept of supply chain resilience. Christopher and Peck (16) defined “supply chain resilience” as “the ability of a supply chain to recover to its original state or a more ideal state after being disrupted.” This concept has also been employed by many scholars to investigate supply chain security management at the enterprise level.

Some scholars have elevated the enhancement of supply chain resilience to the level of national economic strategy, defining it as the ability to flexibly respond to new crises and rapidly recover (17). Currently, most scholars tend to define supply chain resilience from the perspective of dynamic capabilities. According to Larin et al. (18), supply chain development strategies should ensure that each link can efficiently respond to adverse factors. Furthermore, supply chain resilience places greater emphasis on the ability to respond promptly and continuously improve when facing uncertainty and risks (19).

#### 2.2.2 Influencing factors and measurement of supply chain resilience

Research on the factors influencing supply chain resilience has been conducted extensively by scholars, focusing on both internal and external factors. External factors encompass political, social, economic, and environmental aspects. Globalization has driven supply chain resilience to become a national strategy, with social systems and political regimes between countries potentially impacting the flow of capital, information, and logistics within supply chains (20). Wieland (21) noted that uncontrollable factors such as social culture and public opinion have also emerged as major sources of supply chain risk. Compared to external factors, internal factors influencing supply chain resilience are more complex and encompass a broader range of research areas. From an organizational perspective, integration capability (22), collaborative cooperation (23), and communication (24)all positively influence supply chain resilience. From a technological perspective, big data analytics (13) and digital technologies (25) enhance the predictive and management capabilities of supply chains, thereby playing a crucial role in supply chain resilience.

For cross-border e-commerce supply chain resilience, environmental uncertainties—including geopolitical conflicts (e.g., China-U.S. trade frictions), natural disasters (26), and global public health crises (27)—constitute critical disruption factors. Cross-border logistics delays and tariff fluctuations further exacerbate emerging markets’ vulnerability, thereby significantly compromising supply chain resilience. Structurally, supplier diversification (28) enhances resilience through risk dispersion. At the organizational level, joint contingency protocols (29)and cross-cultural management competencies (30)serve as pivotal soft enablers for resilience augmentation.

Existing scholars have primarily constructed supply chain resilience indicators around five main dimensions: prediction, adaptation, response, recovery, and learning, with distinctions made in the sub-dimensions of these five capabilities (31). In terms of research methodologies, many validated and widely applied scales have been used to measure supply chain resilience. However, these scales often rely on subjective assessments and micro-level expressions.

### 2.3 Research of healthcare supply chain

Most existing research on the healthcare industry primarily focuses on technological perspectives, with fewer scholars approaching it from a management science viewpoint. Considering the characteristics of the healthcare industry, it is known for being technology-intensive, knowledge-intensive, patent-dependent, and globally innovative. Consequently, many countries classify the healthcare industry as a strategic emerging industry (32). As a knowledge-intensive industry, the healthcare industry is typically patent-dependent, encompassing pharmaceutical manufacturing, medical technology, and biotechnology. It is characterized by high investment, high risk, high return, and long cycles (33). The majority of current research on the healthcare industry primarily adopts a technological perspective, focusing on cutting-edge fields such as new drug development (34), medical device innovation (35), and breakthroughs in diagnostic and therapeutic technologies (36). However, a limited number of studies have analyzed the healthcare industry from a management standpoint. For instance, Alotaibi and Wilson (37)explored the factors influencing the digital competencies of healthcare professionals, while Ahmed and Hamdan (38) investigated the resilience and digital transformation strategies of healthcare supply chain in emerging economies. These studies provide valuable insights for our subsequent research.

The healthcare supply chain involves numerous participants, particularly in the pharmaceutical supply chain, making its structure relatively complex. Current research on healthcare supply chains primarily focuses on pricing and profit coordination decisions. Zandkarimkhani et al. (39)investigated the design of a perishable pharmaceutical supply chain network under uncertainty. Similarly, Zahiri et al. (40) studied the design of pharmaceutical supply chain networks under uncertainty, considering product perishability and substitutability within the context of sustainable and resilient supply chains. Settanni et al. (41)proposed an improved interactive multi-objective fuzzy programming method to establish and optimize a multi-period, multi-objective pharmaceutical supply chain model, enriching the healthcare system. Benneyan et al. (42) developed optimization strategies for healthcare supply chains and pre-positioning storage locations to address the demand of home healthcare patients experiencing periodic interruptions. Ma et al. (43) examined the quality of work in healthcare supply chains from the perspective of patient benefits and suggested that optimizing quality work can enhance supply chain profitability.

### 2.4 Research commentary

Existing literature indicates that research on the digital economy has achieved significant breadth and depth, systematically exploring its integration with cross-border e-commerce, including business models, development promotion, and supply chain digital transformation. It provides a theoretical foundation for this study.

However, existing studies predominantly adopt a “technology-economy” dualistic analytical framework, failing to adequately reveal the differentiated mechanisms of digital economy in specialized industries like healthcare. A tripartite analytical model integrating industrial characteristics, digital economy, and regional development remains notably absent. Supply chain resilience has emerged as a key research focus in supply chain management, with scholars examining its influencing factors and evaluation systems from multiple perspectives. Nevertheless, current research—particularly on manufacturing enterprises—overwhelmingly emphasizes firm-level analyses, focusing on organizational and operational models while neglecting macro-industrial dimensions and external economic circulation. In healthcare industry research, the predominance of technological perspectives and case-study methodologies has led to excessive focus on individual institutions’ operational optimization. This approach lacks systematic consideration of regional or industry-wide healthcare supply chain resilience, revealing critical gaps in both theoretical frameworks and practical applications.

Therefore, we innovatively develop a supply chain resilience evaluation system for cross-border e-commerce in the healthcare sector from macro-industrial and regional economic perspectives. Methodologically, it enriches healthcare industry research by integrating industrial characteristics, digital economy development, and regional coordination into a unified analytical framework to examine their impacts and mechanisms on supply chain resilience. These theoretical breakthroughs not only expand the application boundaries of cross-border e-commerce supply chain resilience theory, but also provide critical policy implications for the global deployment of healthcare industries in the post-pandemic era.

## 3 Theoretical basis and research hypothesis

### 3.1 Digital economy and resilience of cross-border e-commerce supply chains in healthcare

Driven by the new wave of technological revolution, the digital economy has emerged as a novel economic paradigm characterized by digital technologies (e.g., internet, big data, and artificial intelligence) as its core driver and data as its key production factor. As a product of deep integration between information technology and traditional economies, it fundamentally restructures production, distribution, and consumption patterns through digital transformation. This paradigm significantly influences supply chain operations and production efficiency while creating new opportunities for value generation in supply chain (44). Supply chain resilience emphasizes a system’s ability to rapidly adapt, recover, and continuously optimize in response to internal and external disruptions. Its essence lies in enabling supply chain to achieve higher levels of equilibrium and competitiveness amid uncertainty. The most direct impact of the digital economy on healthcare cross-border e-commerce supply chain manifests in the application of digital technologies, which enhance resilience across four key dimensions: risk early warning, operational flexibility, collaborative efficiency, and compliance management.

From the perspective of risk early warning, big data analytics enables organizations to process vast operational datasets, effectively identifying and assessing risks. This capability improves planning timeliness and facilitates risk mitigation (45, 46). By efficiently integrating and processing information data, digital technologies can help enterprises obtain rapidly changing customer demand information and supplier inventory status information, significantly improving the visibility (47) and traceability (48) of the supply chain. This facilitates precise demand–supply matching and enables dynamic optimization of resource allocation. In addition to integrating information, digital technologies can also facilitate experiential learning from data. Technologies such as blockchain and artificial intelligence enable the digitization and standardization of existing knowledge and experience, providing supply chain members with information, knowledge, and experience to handle disruptive events, thereby enhancing their resilience (49, 50)and enhancing operational management efficiency.

From the perspective of collaborative efficiency, the high innovation, strong penetration, and wide coverage of the digital economy have profoundly changed the total factor productivity at various levels of the real economy (51), driving the development of digital platforms and digital infrastructure. This can overcome the limitations of resource scarcity and homogeneity within cross-border e-commerce and cross-border logistics enterprises (52), accelerate the flow of resources and information between cross-border e-commerce and cross-border logistics, reduce information asymmetry, and significantly improve inefficiencies and unnecessary resource wastage in the supply–demand matching process, thereby lowering the coordination costs between them (53). From a compliance management perspective, the unique regulatory requirements of healthcare products make certification and supervision a critical component in cross-border e-commerce supply chain. The deep integration of digital technologies has significantly enhanced intelligent regulatory capabilities: AI-powered certification engines automatically align products with target market regulations, reducing compliance review cycles and improving audit efficiency; blockchain-based traceability systems enable end-to-end monitoring from raw material procurement to final sales, ensuring each healthcare product meets the quality standards and regulatory requirements of destination markets. This digital regulatory framework not only mitigates compliance risks but also transforms safety control from reactive responses to proactive prevention. By establishing a robust quality assurance mechanism, it provides a foundational safeguard for the sustainable development of healthcare cross-border e-commerce. Therefore, the following hypothesis is proposed:

*Hypothesis 1*: The development of the digital economy can enhance the resilience of the cross-border e-commerce supply chain in healthcare.

### 3.2 Digital economy, foreign trade dependence, and resilience of cross-border e-commerce supply chains in healthcare

Foreign trade dependence on typically refers to the degree to which a country or region’s national economy relies on international trade, reflecting both the depth of its participation in global division of labor and its level of economic openness. From the perspective of the broader developmental environment, China’s marginal contribution of foreign trade to GDP is diminishing, with the growth rate of dependence on foreign trade gradually slowing down, and even showing a downward trend (54). Foreign trade dependence is influenced by multiple factors, including the stage of economic development, industrial structure characteristics, market size, and policy orientation. However, the digital economy era is driving transformative changes: digital technologies are reshaping the traditional mechanisms behind foreign trade dependence by enhancing domestic industrial chain coordination efficiency and accelerating the digital transformation of trade in services. The impact of the digital economy on foreign trade dependence is primarily manifested in two aspects: traditional trade patterns and domestic market influences. Firstly, concerning traditional trade patterns, the digital economy has propelled the development of service trade, whereby digital technologies enable the provision of globalized services across borders. By developing service trade, nations can reduce reliance on goods trade and mitigate risks associated with foreign trade. Simultaneously, through technologies such as the internet, big data, and artificial intelligence, enterprises can achieve intelligent management of supply chains, thereby becoming more independent in production and supply, and reducing reliance on external supply chains. For the domestic market, the permeability, integration, and synergy of the digital economy continuously enhance the digitization level of China’s information and communication industry, which facilitating the domestic circulation. Concurrently, through the digital transformation of industries, it increases the output and efficiency of traditional industries and the real economy, thereby enhancing domestic enterprises’ supply capacity and their ability to accurately match market demand. Additionally, the digital economy has given rise to new consumer formats such as platform economy and sharing economy, creating new consumption patterns and driving continuous expansion in domestic demand (55).

The Resource Dependence Theory posits that organizations must acquire critical resources from external environments to sustain operations, with such dependence creating power dynamics among entities—where the criticality and scarcity of resources determine the bargaining advantage of external actors (56). To mitigate dependency risks, firms adopt strategies like supply diversification, vertical integration, and strategic alliances to reconfigure their resource networks. From the perspective of this theory, lower foreign trade dependence essentially reflects a regional economic system’s deep embeddedness in internal resource endowments and market structures. This inward-oriented resource acquisition pattern drives cross-border e-commerce enterprises in the healthcare sector to construct localized resource pools, thereby reducing reliance on external critical resource providers.

Specifically, in the dimension of resource acquisition, cultivating local supplier ecosystems creates alternative resource nodes, enabling medical cross-border e-commerce firms to overcome the “resource dependence dilemma” (56) by establishing polycentric supply networks to disperse operational risks arising from environmental uncertainty. Secondly, in terms of power balancing, an evenly distributed supply chain structure diminishes the bargaining leverage of suppliers from specific (external) regions, allowing these firms to dynamically adjust procurement strategies based on resource importance and substitutability, thereby achieving a rebalancing of resource control. Finally, regarding organizational adaptability, the implementation of localized management systems essentially responds to the need for “boundary-spanning” (57). Through on-site supply chain teams and infrastructure, healthcare cross-border e-commerce enterprises can more effectively absorb and translate local market knowledge, enhancing their agile responsiveness to fluctuations in cross-border demand. This organizational restructuring, rooted in deepening local resource integration, not only reduces information asymmetry costs in cross-border transactions but also establishes an institutional buffer mechanism for emergencies, ultimately leading to a structural enhancement in the resilience of medical cross-border e-commerce supply chain. Hence, the following hypothesis is proposed:

*Hypothesis 2*: The digital economy enhances the resilience of medical cross-border e-commerce supply chains by reducing foreign trade dependence.

### 3.3 Digital economy, export technological complexity, and resilience of cross-border e-commerce supply chains in healthcare

Global Value Chain (GVC) theory serves as a critical analytical framework for examining the division of labor and cooperation in the global economy, with its core focus on deconstructing and coordinating the entire process—from conceptual design to final consumption—of products or services across different countries and firms. From the perspective of GVC theory, export technological sophistication not only reflects, to some extent, the technological content and production efficiency of exported goods (58), but also indicates a country’s or firm’s position and competitiveness within the GVC division of labor. Some scholars further regard it as a key measure of trade structure optimization (59). A higher level of export technological sophistication indicates that firms within a country are better positioned to occupy strategic, high-value-added segments of the global value chain (GVC), such as R&D and design. The digital economy can significantly enhance the efficiency of information flows and coordination capabilities across GVC segments by reshaping the operational dynamics of global value chains. Specifically, digital technologies break down the spatial and temporal barriers inherent in traditional GVC, reducing information asymmetry and coordination costs for firms engaged in global production networks. This facilitates the cross-border integration of innovation factors across R&D, production, and logistics, thereby driving the technological upgrading of export products (60). Furthermore, the development of the digital economy has facilitated deeper integration in global technology markets. Through digital platforms, firms can more efficiently access cutting-edge international technological knowledge, which stimulates their R&D activities and increases investment in innovation (61), thereby enhancing their competitive advantage in global value chain. The construction of new digital infrastructure not only reduces communication, transaction, and information search costs but also drives trade upgrading through technology spillover effects (62), helping firms break free from the “low-end lock-in” dilemma and achieve upward mobility in GVC. Beyond technological capability, infrastructure, and trade costs, the digital economy also strengthens firms’ absorptive capacity for advanced GVC technologies by improving the digital skills of human capital, providing sustained talent support for the continuous advancement of export technological sophistication.

While high technological complexity may pose challenges in supply chain management, in the healthcare industry, mastery of highly complex export technologies contributes to enhancing product quality and safety. In the global value chain of the healthcare industry, products with high technological sophistication typically correspond to core control segments of the value chain. Such products must comply with stringent international certification standards (e.g., FDA, GMP), and their manufacturing processes involve complex technical procedures and quality control systems. By enhancing export technological sophistication, firms can better meet these international standards, ensuring consistent product quality across all GVC stages—from R&D to final consumption. Particularly in cross-border logistics, the application of advanced temperature-control technologies and digital traceability systems enables firms to monitor product flows in real time throughout the GVC, allowing timely responses to potential quality risks. This capability strengthens the overall resilience and reliability of the value chain. Hence, the following hypothesis is proposed:

*Hypothesis 3*: The digital economy enhances the resilience of healthcare cross-border e-commerce supply chains by elevating export technological complexity.

### 3.4 Digital economy, export trade concentration, and resilience of cross-border e-commerce supply chains in healthcare

Export trade concentration reflects the degree of agglomeration in export commodities or target markets across product categories and geographical dimensions. This concept encompasses two dimensions: product concentration, which measures the extent to which export earnings are concentrated in a limited number of commodity categories; and market concentration, which characterizes the dispersion level of export destinations. High concentration is often associated with comparative advantages in resource endowments or specialized division of labor, while low concentration reflects a diversification strategy. The digital transformation serves as a crucial driving force for enhancing the diversification of enterprise exports (63). External risks contribute to the highly dynamic and complex nature of the international market, making adaptability to new environments a critical capability for enterprises in responding to external dynamic changes. Under the advancement of the digital economy, digital technologies enable organizations to swiftly and accurately capture changes in the external environment (64). By influencing internal organization, production methods, management costs, information dissemination methods, etc., digital technologies endow organizations with acute international market insights and robust resource acquisition capabilities (65), thereby providing favorable conditions for expanding the diversity of international markets. The reduction of trade costs facilitates the formation of new trade connections, thereby lowering the concentration of exports and enhancing the level of export diversification (66). Specifically, the application of digital financial technologies can streamline cross-border trade payment and settlement processes, reducing transaction costs. This enables small enterprises and emerging market countries to more easily participate in international trade, thereby promoting export diversification.

The core proposition of portfolio theory posits that by allocating capital across diversified assets with low return correlations, investors can either minimize risk at a given return level or maximize returns at an acceptable risk threshold. This theoretical framework demonstrates that investment diversification reduces unsystematic risk due to heterogeneous responses of different assets to market shocks. When transposed to international trade, this financial paradigm suggests that export structures essentially constitute “trade portfolios”: export product categories function as distinct assets, while target markets represent alternative investment channels. Excessive concentration in either export products or markets (analogous to “overweight positions” in financial portfolios) may engender export instability (67, 68), exposing trade flows to idiosyncratic risks including demand volatility, technological substitution, or policy barriers that could trigger significant export earnings fluctuations. Conversely, diversification across products and markets has been empirically shown to mitigate export volatility (69), effectively constructing an “efficient trade portfolio” that employs risk-hedging mechanisms to cushion external shocks—when returns from certain products/markets decline, the stable performance of others creates compensatory effects.

For cross-border e-commerce healthcare supply chain, regional diversity in healthcare medical products functions analogously to an investment portfolio holding stocks across different sectors. When specific product categories encounter technical barriers or regulatory scrutiny, other product lines can maintain stable cash flows. This approach not only mitigates market risks but also reduces dependency on particular products or markets. In the event of external shocks affecting certain healthcare products, the performance of alternative export commodities can compensate for losses, thereby enhancing supply chain stability and risk resilience. Moreover, greater export product diversity reflects stronger regional market competitiveness and contributes to sustainable regional development.

Therefore, the following hypothesis is proposed:

*Hypothesis 4*: The development of the digital economy enhances the resilience of healthcare cross-border e-commerce supply chains by reducing export concentration.

**Figure 1:**
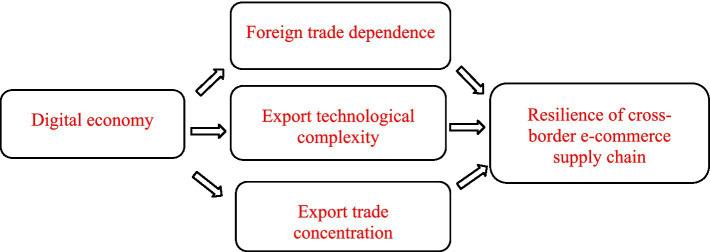
Theoretical model.

Based on the aforementioned theoretical analysis and research hypotheses, the theoretical model diagram of this study is shown in Figure 1.

## 4 Research design

### 4.1 Model assumptions

To examine the impact of the digital economy on the resilience of healthcare cross-border e-commerce supply chains, we set the following baseline model (Equation 1):


(1) $$\mathrm{Rev}l_{\mathrm{it}}=\alpha +\beta_{1}\text{Digd}e_{\mathrm{it}}+\sum_{j}\beta_{j}\text{Contro}l_{\mathrm{it}}+\mu_{i}+\gamma_{t}+\varepsilon_{\mathrm{it}}$$

Where: the subscripts i and t represent provinces and years, respectively; Revl denotes the level of resilience of medical cross-border e-commerce supply chains; Digde represents the level of development of the digital economy; Control represents the control variable group; $\mu_{i}$ and $\gamma_{t}$ represent province fixed effects and time fixed effects, respectively; $\varepsilon_{\mathrm{it}}$ is the random error term. If the regression coefficient $\beta_{j}$ is significantly positive, it indicates a direct positive and significant impact of digital economy development on the resilience of healthcare cross-border e-commerce supply chains.

### 4.2 Variables description

#### 4.2.1 Dependent variable: the level of resilience of healthcare cross-border e-commerce supply chains (Revl)

Currently, the construction of resilience indicators for supply chains typically includes dimensions such as prediction, adaptation, recovery, response, and innovation capabilities. In light of this, this study measures the resilience level of healthcare cross-border e-commerce supply chains primarily from four dimensions: responsiveness, adaptability, recovery, and learning and innovation capabilities, based on the dynamic capability theory. Responsiveness, adaptability, and recovery capabilities mainly refer to the ability of the supply chain to respond to disturbances, while learning and innovation capability pertains to the ability of the supply chain to adapt after disturbances. Corresponding indicators are selected for measurement in each dimension, and principal component analysis is employed to calculate the resilience level of healthcare cross-border e-commerce supply chains for each province and time period.

Recovery capability, as referenced by WEI et al. (65), is primarily reflected by the rate of change in healthcare exports. The numerical fluctuations and trend variations in healthcare exports essentially serve as a “barometer” of the synergistic efficacy across supply chain segments, with their data dynamics precisely capturing the entire process from localized disruptions to systemic recovery within the supply chain. When the fluctuation rate of export volume is low, it indicates that the supply chain can quickly adjust and recover in the face of external shocks or emergencies, demonstrating strong resilience.

Supply chain resistance refers to the ability of the supply chain system to effectively withstand and maintain normal operation when facing external shocks, risks, or changes. The cargo capacity and transportation capability of a region reflect its logistical distribution competence and overall regulatory capacity, which indirectly demonstrates the development level of regional supply chain and their “resilience redundancy” when facing supply chain disruptions. From an ecosystem perspective, the quantitative scale of healthcare enterprises within a region directly determines the nodal density of the supply chain network. This density effect inherently possesses risk dispersion characteristics and innovation multiplier effects, thereby enhancing supply chain flexibility.

Responsiveness refers to the supply chain system’s ability to quickly respond and adjust to changes in market demand, external environmental factors, and emergencies. Inventory functions as an “emergency response reserve pool,” where strategic inventory deployment plays a crucial role in providing operational buffers for supply chain systems. Given the high-value and time-sensitive nature of healthcare products, inventory management must achieve “precision redundancy”—avoiding expiration losses from overstocking while ensuring rapid response during emergencies. This balancing capability serves as a key metric for evaluating supply chain responsiveness. Consequently, the inventory turnover rate of regional healthcare industries reflects the response capacity of cross-border e-commerce healthcare supply chain. Furthermore, a diversified network of collaborative partners establishes “distributed response hubs.” A broader base of partners provides cross-border e-commerce operations with increased resources and alternatives. In scenarios of sudden demand surges or regional supply chain disruptions, the ability to quickly activate alternative partners enables production capacity substitution and logistics channel switching. This mechanism significantly enhances the flexibility and stability of the supply chain.

Learning and innovation capability reflects the strength and endurance of supply chain organizations. Higher levels of innovation indicate stronger technical support for cross-border e-commerce supply chains, as well as more innovation resources and talent in the region. Additionally, it can promote information sharing and collaboration. The development level and innovation capacity of the technological market most directly reflect the knowledge flow and innovation synergy effects within a region. Consequently, we will employ these two dimensions—technological market development and innovation performance—as metrics to evaluate the learning and innovation capabilities of cross-border e-commerce healthcare supply chain. Specific indicators are detailed in Table 1.

**Table 1:** Evaluation indicators for the resilience of healthcare cross-border e-commerce supply chains.

Primary Indicators	Secondary Indicators	Description of Indicators
Recovery Capability	Fluctuation degree of healthcare product export volume	Absolute value of export volume change rate
Resistance Capability	Regional goods carrying capacity	Goods turnover volume
	Scale of cross-border healthcare e-commerce enterprises	Number of cross-border healthcare e-commerce enterprises
	Regional transportation capacity	Freight volume
Responsiveness	Regional healthcare product inventory turnover rate	Sales amount/Average inventory
	Supplier network relationships	Number of bilateral trade partnerships
Learning and Innovation Capability	Level of technological market development	Total value of technology contracts
	Level of innovation	Logarithm of patent applications

#### 4.2.2 Core explanatory variable: level of digital economy development (Digde)

Drawing on the measurement methods proposed by Wang et al. (70) and Zhao et al. (71), a comprehensive evaluation system comprising four primary dimensions—digital infrastructure, digital industrialization, industrial digitalization, and digital innovation capability—and 10 secondary dimensions, totaling 21 detailed indicators, was constructed as shown in Table 2 below. The entropy weight method (entropy value method) was then employed to calculate the level of digital economy development for each time period.

**Table 2:** Composite index of digital economy development.

Primary Indicators	Secondary Indicators	Description of Indicators
Digital infrastructure	Level of internet penetration	Number of internet broadband access ports
		Number of internet broadband access users
		Number of internet domain names
	Level of mobile phone penetration	Density of mobile phone base stations
		Mobile phone penetration rate
	Breadth of information transmission	Length of long-distance optical cables per unit area
Digital industrialization	Software and information technology services industry	Proportion of software business revenue to GDP
		Number of employees in the information transmission, software, and information technology services industry
	Development level of the electronic information manufacturing industry	Proportion of information technology service revenue to GDP
		Proportion of total telecommunications business to GDP
		Per capita total telecommunications business
	Development level of the postal and telecommunications industry	Per capita total postal business
		Volume of express delivery
		E-commerce transaction value of enterprises
Industrial digitalization	Degree of enterprise digitalization	Proportion of enterprises engaged in e-commerce activities
		Number of computers used per 100 employees in enterprises
		Number of websites owned per 100 enterprises
	Digital inclusive finance	Digital inclusive finance index
Digital innovation capability	Level of research and experimental development	Full-time equivalent of R&D personnel in industrial enterprises above designated size
		R&D expenditure of industrial enterprises above designated size
		Number of R&D projects (topics) in industrial enterprises above designated size

Digital infrastructure, as a fundamental indicator, incorporates hard metrics such as internet broadband access and mobile communication coverage (e.g., port capacity, base station density). These parameters directly determine data flow efficiency and connectivity breadth, serving as the physical foundation for digital economy operations. Digital industrialization focuses on scale indicators of the information and communication technology (ICT) sector (e.g., software revenue share, total telecom services), which objectively quantify the maturity and economic contribution of core digital industries. Industrial digitalization is measured through enterprise digital penetration rates (e.g., e-commerce transaction ratio, computer utilization density), reflecting the practical outcomes of traditional industry transformation and revealing the depth of digital technology’s empowerment in the real economy. Digital innovation capability employs R&D intensity indicators (e.g., R&D expenditure, full-time equivalent research personnel), as these directly represent sustainable drivers of technological iteration and constitute core elements for assessing long-term digital economy competitiveness.

The entropy weight method is an objective quantitative approach that automatically calculates indicator weights based on data dispersion characteristics. Compared with subjective methods like expert scoring, it effectively avoids weighting biases. In this study, we first applied range standardization to eliminate dimensional effects. Since all indicators are positively oriented, we standardized them using the Equation 2:


(2) $$X_{\mathrm{ij}}^{\prime }=\frac{x_{\mathrm{ij}}-\min(x_{j})}{\max(x_{j})-\min(x_{j})}$$

Let $x_{\mathrm{ij}}$ represent the value of the j-th indicator for the i-th sample.

Subsequently, we calculate the proportion of each indicator, see Equation 3 for details:


(3) $$p_{\mathrm{ij}}=\frac{x_{\mathrm{ij}}^{,}}{\sum_{i=1}^{n}x_{\mathrm{ij}}^{,}}$$

Where $p\;Z_{\mathrm{ij}}$ ∈ (0,1), and $\sum p_{\mathrm{ij}}=1$ ;

Subsequently, the entropy value for each indicator is determined using the information entropy formula, see Equation 4 for details:


(4) $$e_{j}=-k\sum_{i=1}^{n}p_{\mathrm{ij}}\ln p_{\mathrm{ij}}$$

Where: k = 1/ln(n) serves as the normalization constant;

Finally, the objective weight for each indicator is derived through the weighting formula, followed by a weighted calculation of the composite score, refer to Equation 5:


(5) $$w_{j}=\frac{1-e_{j}}{\sum_{j=1}^{m}(1-e_{j})}$$

#### 4.2.3 Mediating variables

This study focuses on healthcare exports as the research subject, and therefore selects foreign trade dependence (Ftrd), export technological complexity (Expt), and export trade concentration (Dexp) as the mediating variables. Foreign trade dependence (Ftrd) is represented by the ratio of the total import and export volume of a province to the GDP of that province.

The export technological complexity (Expt) is measured following the approach of Hausmann et al. (72), calculating the export technological complexity at the provincial level. Firstly, the export technological complexity at the product level for a given year ( ${\text{Prody}}_{p}$ ) is calculated as the Equation 6:


(6) $${\text{Prody}}_{p}=\sum_{m}\frac{\frac{X_{\mathrm{mp}}}{X_{m}}}{\sum_{m}\frac{X_{\mathrm{mp}}}{X_{m}}}Y_{m}$$

Where ${\text{Prody}}_{p}$ represents the technological complexity of export product p, $Y_{m}$ denotes the per capita GDP of region m, and $X_{\mathrm{mp}}$ and $X_{m}$ represent the export value of product p and the total export value of all products from country m, respectively. Based on this, the export technological complexity at the provincial level in China is further constructed by considering the export structure of each province. The detailed calculation procedure is shown in Equation 7:


(7) $$\mathrm{Exp}t_{\mathrm{it}}=\sum_{c=1}^{n}\frac{X_{\mathrm{ipt}}}{X_{\mathrm{it}}}{\text{Prody}}_{p}$$

Where ${\text{Expt}}_{\mathrm{it}}$ represents the export technological complexity of province i in year t , $X_{\mathrm{ipt}}$ denotes the export value of product p from province i in year t , and $X_{\mathrm{it}}$ represents the total export value of province i in year t .

Export diversification and export trade concentration (Dexp) is measured using the Herfindahl–Hirschman Index (HHI). This index reflects the distribution characteristics of the proportions of traded products and can indirectly indicate the degree of diversification. Consequently, it is utilized by the economics community and government management departments as a measure of diversification. The Herfindahl–Hirschman Index (HHI) is defined as the sum of the squares of the percentages of the total export value for various products relative to the total export value, reflecting the concentration of import and export trade. The specific calculation formula is as the Equation 8:


(8) $$\mathrm{HHI}=\sum_{i=1}^{n}s_{\mathrm{ijt}}^{2};s_{i}=\frac{x_{\mathrm{ijt}}}{x_{\mathrm{git}}}$$

Where $s_{\mathrm{ijt}}$ represents the proportion of the export value of product i from province j in period t to the total export value of province j ; $x_{\mathrm{ijt}}$ denotes the export value of product i from province j in period t , and $x_{\mathrm{git}}$ represents the total export value of province j in period t . The HHI index ranges between 0 and 1, with a smaller index indicating lower export concentration, i.e., greater export diversification. It serves as an inverse indicator of export diversification.

#### 4.2.4 Control variables

Drawing from existing literature and the availability of data, the specific control variables selected for this study include: Human capital level (HC): represented by the average years of education; Level of transportation infrastructure (Inft): represented by the logarithm of highway mileage and the logarithm of total freight volume; Labor level (Labor): represented by the natural logarithm of the number of employed persons; Level of industrial structure upgrading (Inds): represented by the logarithm of the ratio of the value added of the tertiary industry to the secondary industry; Government fiscal expenditure (Gov): represented by general government expenditure; Level of industrialization (Indu): represented by the ratio of industrial output value to regional total output value.

### 4.3 Data source

Data pertaining to export transactions are sourced from the customs database, which comprises complete sample data from customs ports, covering detailed information from all export regions. Export technological complexity and the resilience of the cross-border healthcare e-commerce supply chain, calculated using customs data, are considered highly reliable. Given the focus of this study on the healthcare industry, which mainly encompasses pharmaceutical manufacturing and medical devices, export product inquiries primarily involve selected products from these two categories. Other indicators related to the development of the digital economy and resilience assessments are primarily sourced from the “China Statistical Yearbook” and various provincial and local statistical yearbooks. As pharmaceutical manufacturing and medical devices are part of China’s high-tech industries, some data are also obtained from the “China High-Tech Industry Statistical Yearbook.” For the limited missing data, interpolation methods were employed for imputation, while logarithmic transformations were applied to specific variables (e.g., Human capital level) to normalize distributions.

## 5 Empirical analysis

### 5.1 Benchmark regression

Table 3 presents the benchmark regression results for the levels of digital economy and resilience of the healthcare cross-border e-commerce supply chain. Column (1) reports the regression results of the univariate relationship between the digital economy and the resilience of the healthcare cross-border e-commerce supply chain after controlling for bidirectional fixed effects. Columns (2) and (3) display the regression results after including control variables and alternatively incorporating time fixed effects and provincial fixed effects. Column (4) reports the regression results after including control variables and simultaneously incorporating both fixed effects. The results indicate that in Column (4), the coefficient for digital economy development (Digde) is 4.818 and statistically significant at the 1% level. This suggests that digital economy development significantly positively influences the resilience of the healthcare cross-border e-commerce supply chain. Moreover, coefficients in Columns (1) to (4) are all positively significant at the 1% level, confirming Hypothesis 1. The variation in the coefficient of digital economy development across Columns (1) to (4) indicates that the extent to which the digital economy promotes the resilience of the healthcare cross-border e-commerce supply chain is influenced by the year and province. This variability may be attributed to policy differences among provinces and external shocks in different years, but overall, it does not affect the significance of the results.

**Table 3:** Benchmark regression results.

Variables	(1)	(2)	(3)	(4)
	Revl	Revl	Revl	Revl
Digde	4.818*** (10.00)	1.466*** (3.13)	3.669*** (6.68)	2.842*** (4.99)
HC		1.865*** (3.00)	2.043*** (3.06)	0.801 (0.93)
Inft		1.257*** (9.53)	1.169*** (9.62)	1.298*** (8.92)
Labor		−0.204 (−0.84)	0.037 (0.23)	−0.197 (−0.73)
Inds		−0.002 (−0.24)	0.000 (0.02)	0.001 (0.07)
Gov		0.000*** (5.20)	0.000*** (3.66)	0.000*** (3.88)
Indu		0.346 (0.78)	0.244 (0.57)	−0.002 (−0.00)
_cons	2.177*** (38.14)	−15.218*** (−5.72)	−16.428*** (−8.85)	−13.262*** (−4.80)
FE	Yes	No	Yes	Yes
TE	Yes	Yes	No	Yes
N	300	300	300	300
R2	0.700	0.764	0.7872	0.793

*, **, and *** denote significance levels at 10, 5, and 1%, respectively. Values in parentheses represent t-statistics. The same applies to the subsequent tables.

### 5.2 Robustness tests

We examined the robustness of the research results using five methods: replacing the core explanatory variable, adding control variables, lagging one period, GMM model test and conducting endogeneity tests.

#### 5.2.1 Replacing the core explanatory variable, adding control variables and lagging one period

Since this paper uses the entropy method to measure the level of digital economic development, the principal component analysis (PCA) method will be employed in this section to reassess the development level of the digital economy. First, data standardization preprocessing is required to transform the original indicators with different units into standard scores with a mean of 0 and a standard deviation of 1, thereby eliminating the impact of unit differences on the analysis results. The second step involves constructing a correlation coefficient matrix. By calculating the degree of correlation between each indicator, highly correlated indicator groups are identified. The third step is eigenvalue decomposition, where the eigenvalues and eigenvectors of the correlation coefficient matrix are solved. The size of the eigenvalues represents the ability of each principal component to explain the variation in the original data. The fourth step determines the number of principal components. We select the first few principal components with eigenvalues greater than 1 and a cumulative variance contribution rate exceeding 85%, which significantly reduces dimensionality while retaining most of the effective information. Finally, the comprehensive score is calculated by performing a weighted summation of the standardized data based on the variance contribution rate of each principal component. The recalculated digital economy development levels of each province are denoted as Digde_new.

And the added control variable is the level of economic development (Eco). Additionally, we lagged the level of digital economy development by one period (L. Digde). The results are presented in Table 4, which shows that the coefficients for the level of digital economy development are consistent with the benchmark regression results, passing the robustness test.

**Table 4:** Robustness test results.

Variables	(1)	(2)	(3)
	Replacement of the Core Explanatory Variable	Addition of Control Variable	Lagging by One Period
Digde _new	0.432*** (5.25)		
Digde		2.899*** (5.07)	
L. Digde			2.533*** (3.89)
HC	0.689 (0.81)	0.751 (0.87)	−0.121 (−0.13)
Inft	1.235*** (8.52)	1.297*** (8.91)	1.321*** (8.22)
Labor	−0.067 (−0.25)	−0.218 (−0.80)	−0.134 (−0.46)
Inds	0.001 (0.13)	0.001 (0.08)	−0.002 (−0.27)
Gov	0.000*** (7.56)	0.000*** (3.70)	0.000*** (3.44)
Indu	0.018 (0.04)	0.176 (0.37)	−0.236 (−0.47)
Eco		0.093 (1.06)	
_cons	−13.532*** (−4.92)	−14.034*** (−4.91)	−11.900*** (−3.86)
FE	Yes	Yes	Yes
TE	Yes	Yes	Yes
N	300	300	270
R2	0.795	0.794	0.768

#### 5.2.2 GMM model test

To eliminate the impact of endogeneity, the basic panel model was transformed into a system GMM model for re-estimation. In this model, we treated the dependent variable lagged by 1–2 periods as endogenous variables, while the independent variables lagged by one period and all control variables were treated as exogenous variables. The regression results of the GMM model are shown in Table 5. The regression coefficient of “Digde” remains significantly positive at the 1% level, consistent with the results of the basic panel model. Moreover, the AR2 is greater than 0.05, failing to reject the null hypothesis of no second-order autocorrelation in the residuals, and the Hansen value is also greater than 0.1, failing to reject the null hypothesis of valid instrumental variables. This indicates that the GMM model is valid, and the earlier analysis results remain robust.

**Table 5:** GMM model test results.

Variables	(1) Revl
L. Revl	0.593*** (5.13)
Digde	3.453*** (3.83)
HC	0.233 (0.32)
Inft	0.374* (1.78)
Labor	0.024 (0.12)
Inds	0.009 (0.94)
Gov	0.000 (0.68)
Indu	0.516 (0.74)
Constant	−4.761 (−1.60)
FE	Yes
TE	Yes
AR1	0.000
AR2	0.225
Hansen	0.137

#### 5.2.3 Endogeneity test

To avoid biases in the estimated results due to endogeneity, we followed the approach of HUANG et al. (73) and Zhao et al. (71) by selecting the interaction term of the total volume of postal and telecommunications services in 1984 and the internet usage rate in each province from the previous year as the instrumental variable, denoted as Iv. The specific regression results are shown in Table 6. The Anderson canonical correlation LM statistic *p*-value for the instrumental variable Iv is significant at the 1% level, rejecting the null hypothesis of insufficient identification of the instrumental variable. The Cragg-Donald Wald F statistic is greater than the critical value for the Stock-Yogo test at the 1% significance level, thus also rejecting the null hypothesis of weak instruments. The endogenous variables correspond one-to-one with the instrumental variables, so there is no issue of over-identification of the instrumental variable.

**Table 6:** Endogeneity test results.

Variables	(1) First Stage Digde	(2) Second Stage Revl
Digde		2.7136** (2.10)
LnIv	−0.1191*** (−7.08)	
Constant	1.9200*** (5.09)	−12.2514*** (−4.86)
Controls	Yes	Yes
FE	Yes	Yes
TE	Yes	Yes
Anderson canon. corr. LM statistic	49.449***	
Cragg-Donald Wald F statistic	50.129 [16.38]	
Observations	300	300
R-squared		0.987

*, **, and *** denote significance levels at 10, 5, and 1%, respectively. Values in parentheses are t-statistics. Values within curly brackets are critical values for significance levels of the Stock-Yogo test.

## 6 Mechanism tests

We will further examine the underlying mechanism through which the digital economy influences the resilience of the healthcare cross-border e-commerce supply chain, and analyze the impact pathways of different dimensions of the digital economy on the resilience of the healthcare cross-border e-commerce supply chain. Specifically, we will first examine the impact of the digital economy development level on foreign trade dependence, export technological complexity, and export diversification. Then, we will separately examine the impact of these three dimensions on the resilience of the healthcare cross-border e-commerce supply chain. Finally, all dimensions and the level of digital economy development will be included in the regression model to examine the changes in the coefficient and significance of the impact of the digital economy development level on supply chain resilience. The specific model is as follows:


(9) $$\mathrm{Rev}l_{\mathrm{it}}=\alpha +\beta_{1}\text{Digd}e_{\mathrm{it}}+\sum_{j}\beta_{j}\text{Contro}l_{\mathrm{it}}+\mu_{i}+\gamma_{t}+\varepsilon_{\mathrm{it}}$$

(10) $$\mathrm{me}d_{\mathrm{it}}=\alpha +\beta_{1}\text{Digd}e_{\mathrm{it}}+\sum_{j}\beta_{j}\text{Contro}l_{\mathrm{it}}+\mu_{i}+\gamma_{t}+\varepsilon_{\mathrm{it}}$$

(11) $$\mathrm{Rev}l_{\mathrm{it}}=\alpha +\beta_{1}\mathrm{me}d_{\mathrm{it}}+\beta_{2}\text{Digd}e_{\mathrm{it}}+\sum_{j}\beta_{j}\text{Contro}l_{\mathrm{it}}+\mu_{i}+\gamma_{t}+\varepsilon_{\mathrm{it}}$$

Model 9 investigates the relationship between the digital economy and the resilience of the healthcare cross-border e-commerce supply chain. Model 10 considers the relationship between the mediator variable and the digital economy. Model 11 examines the joint effects of the digital economy and the mediator variable on the resilience of the cross-border medical e-commerce supply chain. “med” represents the mediator variable, which includes foreign trade dependence (Ftrd), export technological complexity (Expt), and export diversification (Dexp).

### 6.1 Mediation mechanism test

The regression results are shown in Table 7. Columns (1), (2), and (3) of Table 7 indicate that the estimated coefficients are significantly correlated, and the signs of the coefficients suggest that the development of the digital economy can reduce dependence on foreign trade, ultimately enhancing the resilience of the healthcare cross-border e-commerce supply chain. These results indicate that reducing dependence on foreign trade is one of the mediating channels through which the digital economy influences the resilience of the healthcare cross-border e-commerce supply chain, thereby validating Hypothesis 2.

**Table 7:** Mechanism test results.

Variables	(1)	(2)	(3)	(4)	(5)	(6)	(7)
	Revl	Ftrd	Revl	lnExpt	Revl	Dexp	Revl
Digde	2.842*** (4.99)	−0.977*** (−7.17)	2.356*** (3.79)	−0.205** (−2.50)	3.184*** (5.68)	−0.444*** (−3.10)	2.535*** (4.43)
Ftrd			−0.498* (−1.91)				
lnExpt					1.672*** (3.95)		
Dexp							−0.692*** (−2.81)
_cons	−13.262*** (−4.80)	1.579** (2.39)	−12.476*** (−4.49)	8.404*** (21.09)	−27.311*** (−6.13)	−0.547 (−0.79)	−13.641*** (−5.00)
Controls	Yes	Yes	Yes	Yes	Yes	Yes	Yes
FE	Yes	Yes	Yes	Yes	Yes	Yes	Yes
TE	Yes	Yes	Yes	Yes	Yes	Yes	Yes
N	300	300	300	300	300	300	300
R2	0.793	0.433	0.796	0.980	0.805	0.122	0.799

Columns (1), (6), and (7) of Table 7 show that the estimated coefficients are significant at the 1% level. The signs of these coefficients imply that the development of the digital economy can reduce the concentration of export products, thereby increasing export diversity, which ultimately enhances the resilience of the healthcare cross-border e-commerce supply chain. These findings indicate that export product concentration is one of the mediating channels through which the digital economy contributes to the resilience of the healthcare cross-border e-commerce supply chain, validating Hypothesis 4.

Results from Columns (4) and (5) of Table 7 demonstrate that after introducing the mediating variables, the coefficient of the core explanatory variable increases, and the coefficient of the mediating variable is inversely related to that of the core explanatory variable. According to the explanation by WEN and YE (74), this situation can be attributed to a certain degree of masking effect. Specifically, export technology complexity plays a masking role between the level of digital economy development and the resilience of the healthcare cross-border e-commerce supply chain, obscuring the direct impact of digital economy development on the resilience of the healthcare cross-border e-commerce supply chain. However, whether it is a mediating effect or a masking effect, it indicates an indirect effect exists between export technology complexity, the digital economy, and the resilience of the healthcare cross-border e-commerce supply chain. In other words, the development of the digital economy can promote the resilience of the healthcare cross-border e-commerce supply chain by enhancing export technology complexity, thus validating Hypothesis 3.

### 6.2 Sobel test and bootstrap test

To strengthen the verification of the mediation mechanism, we conducted further tests: the Sobel test and the Bootstrap test. As shown in Table 8, the Sobel test results indicate that the Sobel statistic for the mediation path of Mediating Mechanism 1 (“digital economy—foreign trade dependence—healthcare cross-border e-commerce supply chain resilience”) is not significant at any conventional level. In contrast, the Sobel statistics for the other two mediation paths are significant at the 10% level. This suggests that Hypothesis 2 is not supported by the Sobel test, which deviates from the earlier three-step mediation test results. The Sobel test assumes that the sampling distribution of the mediation effect approximates normality. However, in practice—especially in small samples—the distribution of the product term is often skewed, which can lead to biased test results. Therefore, the Bootstrap method provides greater accuracy and robustness in assessing mediation effects.

**Table 8:** Sobel test results.

Measurement items	Mediating Mechanism 1	Mediating Mechanism 2	Mediating Mechanism 3
Sobel Value	0.486 (1.843)	−0.348* (−2.150)	0.307* (2.082)
Mediating Effect Coefficient	0.486 (1.843)	−0.348* (−2.150)	0.307* (2.082)
Direct Effect Coefficient	2.356*** (3.791)	3.190*** (5.6984)	2.535*** (4.428)
Total Effect	2.842*** (4.990)	2.842*** (5.004)	2.842*** (3.791)
Mediation Effect Ratio	17.109%	−10.914%	12.120%

*, **, and *** denote significance levels at 10, 5, and 1%, respectively. Values in parentheses are t-statistics.

As shown in Table 9, the Bootstrap test results indicate that neither the direct nor indirect effects of the three mediation mechanisms include zero within the 95% confidence interval, rejecting the null hypothesis at valid significance levels. This confirms the existence of mediation (or suppression) effects, providing further support for Hypotheses 2, 3, and 4. Based on both the Sobel and Bootstrap test results, the following conclusions can be drawn: foreign trade dependence mediates the relationship between the digital economy and healthcare cross-border e-commerce supply chain resilience, accounting for 17.11% of the total effect; export technological complexity exhibits a suppression effect (as the signs of the indirect and direct effects are opposite), with the suppression effect amounting to 10.91% (the absolute value of the ratio of the indirect effect to the direct effect); export product concentration mediates the relationship between the digital economy and healthcare cross-border e-commerce supply chain resilience, explaining 12.12% of the total effect.

**Table 9:** Bootstrap test results.

Mechanisms	Effect category	Observed coefficient	Bias	Bootstrap std. err.	[95% conf. interval]
Mediating Mechanism 1	Indirect	0.4861913	−0.0647742	0.25446265	(0.0531118, 1.16549)
	Direct	2.355598	−0.038791	0.84588663	(0 0.6913589,4.035667)
Mediating Mechanism 2	Indirect	−0.34247298	−0.003599	0.18389279	(−0.8521886, −0.0719047)
	Direct	3.1842618	−0.0020825	0.85674249	(1.5771,4.889731)
Mediating Mechanism 3	Indirect	0.30720395	−0.0096761	0.15284604	(0.093417,0.7069903)
	Direct	2.5345849	−0.000544	0.87673567	(0.8889207,4.353372)

## 7 Heterogeneity analysis

### 7.1 Regional heterogeneity

Geographical location can influence the economic development of a region. Areas located at transportation hubs or rich in resources are generally more favourable for economic development, attracting investment and commercial activities. This study divides the full sample into eastern and central regions versus the western region for analysis. The results are shown in Columns (1) and (2) of Table 10: the impact coefficient of the digital economy on the resilience of the healthcare cross-border e-commerce supply chain in the western region (3.979) is greater than that in the eastern and central regions (2.021), indicating a more pronounced positive enabling effect of the digital economy in the western region.

**Table 10:** Heterogeneity analysis results.

Variables	(1)	(2)	(3)	(4)
	Eastern and Central Regions	Western Region	Pre-Policy Implementation	Post-Policy Implementation
Digde	2.021*** (2.88)	3.979*** (2.65)	0.856 (0.38)	1.955*** (2.88)
_cons	−21.842*** (−5.65)	2.180 (0.50)	−7.579 (−0.95)	−12.231*** (−3.43)
FE	Yes	Yes	Yes	Yes
TE	Yes	Yes	Yes	Yes
N	190	110	90	210
R^2^	0.831	0.814	0.632	0.741

China’s eastern and central regions are situated at the nation’s economic centre, which facilitate logistics and the movement of people, featuring developed transportation networks and convenient transportation conditions. Additionally, these regions are densely populated, offering vast potential for commercial and market development. These inherent conditions have allowed the digital economy in China’s eastern and central regions to start earlier and develop to a relatively higher level, significantly enhancing the positive promotive effects of the digital economy. In recent years, China has been vigorously implementing the “Western Development” strategy. Compared to the relatively saturated state of digital economy development in the eastern and central regions, the western region is still in the stage of increasing marginal returns from digital economy development. Therefore, the western region can more significantly absorb the positive impact of the digital economy on the resilience of the healthcare cross-border e-commerce supply chain.

### 7.2 Policy heterogeneity

Since 2015, China has established comprehensive pilot zones for cross-border e-commerce. These zones serve as pioneering areas aimed at exploring the technical standards, business processes, regulatory models, and informatization construction of various aspects of cross-border e-commerce. To assess the effectiveness of these comprehensive pilot zones, this study regards 2015 as a watershed year and divides the sample into periods before and after the implementation of policies. The results, as shown in Columns (3) and (4) of Table 10, indicate that the impact of the digital economy on the resilience of the healthcare cross-border e-commerce supply chain was not statistically significant before the establishment of comprehensive pilot zones for cross-border e-commerce. However, after the establishment of these zones, the digital economy significantly promotes the resilience of the healthcare cross-border e-commerce supply chain. This suggests that the establishment of comprehensive pilot zones for cross-border e-commerce has facilitated the role of the digital economy in enhancing the resilience of the healthcare cross-border e-commerce supply chain, providing strong support and impetus for the development of the healthcare cross-border e-commerce industry.

## 8 Conclusions and policy recommendations

This study utilizes provincial-level panel data from China to investigate the impact of the digital economy on the resilience of the cross-border e-commerce supply chain in the healthcare industry and its underlying mechanisms. Research demonstrates that digital economy development significantly enhances the resilience of healthcare cross-border e-commerce supply chain through three key mechanisms: reducing foreign trade dependence, increasing export technology complexity, and optimizing export concentration. This effect is particularly pronounced in western regions, with cross-border e-commerce comprehensive pilot zones further amplifying the impact. Policy recommendations focus on four dimensions: Industrial integration calls for government policies supporting big data and AI applications in supply chain optimization, along with establishing industry-academia-research platforms for digital solutions. Trade optimization advocates implementing the “dual-circulation” strategy with tax incentives and R&D subsidies to stimulate innovation, complemented by export credit insurance to mitigate risks. Regional coordination emphasizes prioritizing digital infrastructure in western China and establishing east–west collaboration mechanisms for global value chain integration. Institutional innovation recommends replicating successful pilot zone experiences, advancing “single-window” systems for streamlined customs, and encouraging policy experimentation. These measures will effectively foster synergistic development between the digital economy and healthcare cross-border e-commerce.

## Data Availability

The original contributions presented in the study are included in the article/Supplementary material, further inquiries can be directed to the corresponding author.
